# Supporting Physiological Trait for Indirect Selection for Grain Yield in Drought-Stressed Popcorn

**DOI:** 10.3390/plants10081510

**Published:** 2021-07-23

**Authors:** Samuel Henrique Kamphorst, Gabriel Moreno Bernardo Gonçalves, Antônio Teixeira do Amaral Júnior, Valter Jário de Lima, Kátia Fabiane Medeiros Schmitt, Jhean Torres Leite, Valdinei Cruz Azeredo, Letícia Peixoto Gomes, José Gabriel de Souza Silva, Carolina Macedo Carvalho, Gabrielle Sousa Mafra, Rogério Figueiredo Daher, Eliemar Campostrini

**Affiliations:** Plant Breeding Laboratory, Agricultural Science and Technology Center, North Fluminense State University, Av. Alberto Lamego 2000, Campos dos Goytacazes 28013-602, Brazil; samuelkampho@hotmail.com (S.H.K.); gabriel.agrobio@gmail.com (G.M.B.G.); kmedeirosschmitt@gmail.com (K.F.M.S.); torresjhean@gmail.com (J.T.L.); azeredovc@hotmail.com (V.C.A.); leticiap.gomes@hotmail.com (L.P.G.); jgabrielshs@gmail.com (J.G.d.S.S.); carolinamacedocarvalho@gmail.com (C.M.C.); gabrielle.smafra@yahoo.com.br (G.S.M.); rogdaher@uenf.br (R.F.D.); campostenator@gmail.com (E.C.)

**Keywords:** leaf greenness index, normalized difference vegetation index, SPAD, drought, multiple regressions

## Abstract

The identification of traits associated with drought tolerance in popcorn is a contribution to support selection of superior plants under soil water deficit. The objective of this study was to choose morphological traits and the leaf greenness index, measured on different dates, to estimate grain yield (GY) and popping expansion (PE), evaluated in a set of 20 popcorn lines with different genealogies, estimated by multiple regression models. The variables were divided into three groups: morpho-agronomic traits—100-grain weight (GW), prolificacy (PR), tassel length (TL), number of tassel branches, anthesis-silking interval, leaf angle (FA) and leaf rolling (FB); variables related to the intensity of leaf greenness during the grain-filling period, at the leaf level, measured by a portable chlorophyll meter (SPAD) and at the canopy level, calculated as the normalized difference vegetation index (NDVI). The inbred lines were cultivated under two water conditions: well-watered (WW), maintained at field capacity, and water stress (WS), for which irrigation was stopped before male flowering. The traits GY (55%) and PE (28%) were most affected by water restriction. Among the morpho-agronomic traits, GW and PR were markedly reduced (>10%). Under dry conditions, the FA in relation to the plant stalk tended to be wider, the FB curvature greater and leaf senescence accelerated (>15% at 22 days after male flowering). The use of multiple regression for the selection of predictive traits proved to be a useful tool for the identification of groups of adequate traits to efficiently predict the economically most important features of popcorn (GY and PE). The SPAD index measured 17 days after male flowering proved useful to select indirectly for GY, while, among the morphological traits, TL stood out for the same purpose. Of all traits, PR was most strongly related with PE under WS, indicating its use in breeding programs. The exploitation of these traits by indirect selection is expected to induce increments in GY and PE.

## 1. Introduction

In agricultural crops, soil water restriction, caused by the irregular distribution of rainfall during the growth and development of agricultural crops, has a strong impact on yield [1,2,3,4]. Yield losses caused by drought events have become recurrent [5,6,7] and to find measures to minimize them is a challenge nowadays for global agribusiness and, in particular, for Brazil, where agriculture is a key component of the economy [8]. The impact of water restriction depends on the intensity and duration of a dry spell, as well as on the phenological stage of the plant when it occurs [1]. In maize, the grain yield was strongly reduced when the water stress coincided with the phenological stages flowering and grain filling [1,9].

Although grain yield (GY) is usually the target trait by which the degree of drought tolerance is assessed, traits correlated to GY, with greater heritability and easier to measure, are an option to facilitate the selection for plants under water stress [1,10,11]. Under water deficit, direct selection for GY has proved inefficient, since trait heritability is reduced by a high proportion of genetic/environmental variance [3,12,13,14]. Therefore, to cultivate maize on dry soil, morph-physiological traits that are easily determinable and strongly correlated with GY have been sought [1,14,15,16,17,18,19,20]. In the popcorn breeding scenario where grain quality must be evaluated together with GY, genetic advances require gains for grain quality yield and popping expansion (PE) simultaneously [20,21,22], as these are the most important traits for crops.

Maize germplasm with a tolerance for water deficit in the field was evaluated based on the main secondary, GY-related agronomic traits, namely: the shortest interval between male and female flowering [11,13,14,23], delayed leaf and stem senescence (stay-green trait) [13,24,25], greater highest prolificacy [13,26] and lowest number of tassel branches [13,27]. In the mechanism of water stress tolerance, the weakest leaf rolling (related to the highest turgor) and widest angle of leaf inclination in relation to the soil stem (lowest interception of solar radiation at the time of highest air temperature) also play a decisive role [1,27]. Specifically in popcorn and under dry conditions, the trait number of grains per row proved important to identify more productive genotypes with greater popping expansion [20]. The expansion process is associated with the presence of moisture in the grain’s starch granules. When heated (≈180 C), the moisture exerts pressure on the pericarp, and the rupture will expose the endosperm [28]. It means that water shortage during grain formation can affect the physicochemical properties and, therefore, the grain’s capacity to expand. Moreover, no morphological or chemical traits that could explain this phenomenon have been recorded to date [20].

The stay-green genotypes, with later senescence than the average of the standard genotypes, are characterized as the most productive and considered adapted to drought stress [3,12,15,17,24,29,30,31]. In the post-flowering period, late leaf senescence contributes to better grain filling due to the greater transport of photoassimilates to the grains at the end of the filling stage [25]. Methods to assess leaf senescence based on the spectral properties of leaves have been used, i.e., measurements of leaves with a portable chlorophyll meter (SPAD index) and an equipment that determines the normalized difference vegetation index (NDVI index) (spectral vegetation indices), as well as the canopy [3,32]. However, the effectiveness of phenotyping methodologies for popcorn lies partly in the possibility of applying them at the critical stages of grain production [1,15,17] and popping expansion [8,22,33,34].

For maize, different statistical approaches have been used to identify the relationship between the morpho-agronomic traits, as well as those calculated as leaf spectral properties, namely: simple correlation [35], path analysis [36], GT-Biplot [18] and canonical correlation [8]. However, with the exception of simple linear correlation, these methods include the analysis of irrelevant variables for the model, which interferes with the assumptions for the predictors.

Thus, the following scientific question arise: (i) Which of the trait(s) associated with drought stress is (are) most appropriate for the indirect selection of drought-tolerant popcorn germplasm? The objective of this study was to select spectral vegetation indices, such as the greenness index (SPAD) and the Normalized Difference Vegetation Index (NDVI) of the canopy in popcorn lines from different geographic origins in the Americas, to predict the grain yield and popping expansion using model selection based on multiple regression.

## 2. Results

### 2.1. Effects of Different Water Conditions on Dependent (GY and PE) and Morphological Variables and on SPAD and NDVI Values

In a comparison between the water conditions WS and WW, the dependent variables grain yield (GY) and popping expansion (PE) were reduced by 55.30% and 28.75%, respectively (Table 1). Under WW conditions, the mean grain yield was 2548.07 kg ha^−1^ compared to 1139.11 kg ha^−1^ under WS. In the above order of water conditions (WC), the mean PE was 29.35 mL g^−1^ and 20.91 mL g^−1^, respectively (Table 1). In the combined analysis, the effect of interaction G*WC on GY and PE was significant (Table 1).

Water stress induced significant decreases (>10.00%) in the morphological traits 100-grain weight (GW) (23.49%), prolificacy (PR) (15.84%) and delayed the anthesis-silking interval (ASI) (25.44%) (Table 1). In general, the tassel length (TL) (3.50%) and number of tassel branches (TB) (7.01%) tended to decrease, whereas the leaf angle (FA) (8.70%) and leaf rolling (FB) (4.49%) tended to increase in the comparisons between the WCs (Table 1). In the combined analysis, no morphological trait was significantly influenced by the G*WC interaction.

In the comparison of the WS and WW conditions, the SPAD index, evaluated from the 7th to the 42nd day after male flowering, decreased significantly (>17.00%) as of the 22nd evaluation day (SPAD22), and in the evaluation 42 days after male flowering (DAA), the SPAD index decreased by 52.20% (SPAD42). In the initial evaluations of the SPAD index (7, 12 and 17 DAA), the reductions between WCs were less than 11.00%. In the combined analysis, regardless of the measurement date, the SPAD index was not significantly affected by the G*WC interaction (Table 1).

A comparison of the WS and WW conditions for the Normalized Difference Vegetation Index (NDVI), between the 8th and the 38th day after male flowering, detected a significant reduction (>17.00%) from the evaluation on 24 DAA (NDVI24) onwards. In the evaluation 38 days after male flowering (NDVI38), the NDVI was reduced by 33.26%. In the initial NDVI assessments (eight and 19 DAA), the reductions between WCs were less than 10%. In the combined analysis, regardless of the measurement date, the NDVI was not significantly affected by the G*WC interaction (Table 1).

### 2.2. Multiple Regressions Based on Morphological Characters and Dependent Variables GY and PE

Among the morpho-agronomic traits, the variables GW and TB were not significant for any of the tested models (Table 2). Prolificacy was considered significant as an explanatory variable for PE in the models under both WCs but with opposite sign coefficients. The characteristics ASI and FA had significant effects on the response variable PE under WW, and the TL trait was significant in explaining GY under both WCs, as well as PE under WS. However, in the model with the response variable PE, under WS, the sign of the significance coefficient of TL was opposite to that estimated for GY. The trait FB was significant for GY and PE under WS with same-sign coefficients.

### 2.3. Multiple Regressions Based on the SPAD Index of Different Measurement Dates and the Dependent Variables GY and PE

The multiple linear regressions for the different measurement dates of the SPAD index detected significance for only SPAD17 and SPAD42 in at least one of the models (Table 3). The SPAD index assessed 17 days after flowering (SPAD17) was significant as a predictor of GY under WW, while the SPAD index 42 days after flowering (SPAD42) was significant as a predictor of PE under WS (Table 3).

### 2.4. Multiple Regressions Based on NDVI Values of Different Measurement Dates and on the Dependent Variables GY and PE

The effects of the NDVI assessments were only significant for the dependent variable PE. The NDVI evaluation dates with significant effects were 8 days after flowering (NDVI8) under WW, 30 days after flowering (NDVI30), both under WS and WW but with opposite signs and 38 days after flowering (NDVI38) under WS (Table 4).

### 2.5. Biplot Using Significant Traits on Response Variables GY and PE

**Figure 1:**
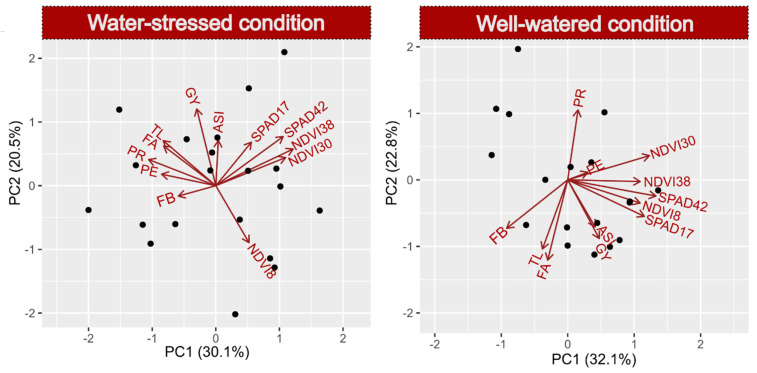
PCA-biplot constructed with traits with significant influence on the response variable grain yields (GY) and popping expansions (PE), selected by multiple regression analyses of the morphological traits (PR = prolificacy, TL = tassel length, TB = number of tassel branches, ASI = anthesis-silking interval, FA = leaf angle and FB = leaf rolling); SPAD index (17 and 42 days after male flowering) and Normalized Difference Vegetation Index (8, 30 and 38 days after male flowering), estimated for 20 popcorn inbred lines under water stress and well-watered conditions.

The PCA-biplots generated for each WC, including the six characteristics selected from the 12 calculated multiple linear regressions (MLR) and added to the dependent variables (PE and GY), showed a similar behavior pattern for GY under both WCs, i.e., the behavior of these characteristics was little influenced by the WC (Figure 1). For PE, the variables FA, TL and FB, which were positively related under WS, had an opposite relationship under WW conditions. The same was observed for SPAD17 and SPAD42 but in the opposite order of signs. Under both WCs, variable PR was associated with PE (Figure 1). The principal component analysis (PCA) under WS had an explanatory power of 50.6%, similar to that under the WW conditions, with an explanatory power of 52.9% (Figure 1).

### 2.6. Selection of Predictors by MLR Based on Pre-Selected Variables

For the second stage of variable selection, including only the selected predictors of each group of variables, the multicollinearity of the model for each WC, as well as error dispersion, was checked with the dependent variables GY and PE (Figure S2).

For GY, the independent variables selected for their significance were FB, TL and SPAD17 (Table 1, Table 2 and Table 3). In the models with the dependent variable GY, the variance inflation factors were close to 1.2, i.e., no variable had to be removed. For PE, the independent variables selected for their significance were PR, TL, ASI, FA, FB, SPAD42, NDVI8, NDVI30 and NDVI38 (Table 1, Table 2 and Table 3). In models with PE as the dependent variable, the variance inflation factor of variable NDVI38 was 11.5 under WS and was pre-eliminated by the Akaike criterion prior to stepwise selection [37]. After the elimination of NDVI38, the variance inflation factors dropped to less than 3.3 for all variables. For error distribution, no evidence of correlation or nonlinearity was detected in any of the four models.

### 2.7. Selection of Model 1—GY under WS

Model 1, where GY was the dependent variable under WS and the variables FB, TL and SPAD17 were used as predictors, maintained the initial model, with an Akaike value of 231.67, adjusted R^2^ of 0.49 and *p* = 0.002 (Final Model 1: GY~FB + TL + SPAD17) and with a significance of only TL in the *t*-test (*p* = 0.05) in the multiple regression.

**Figure 2:**
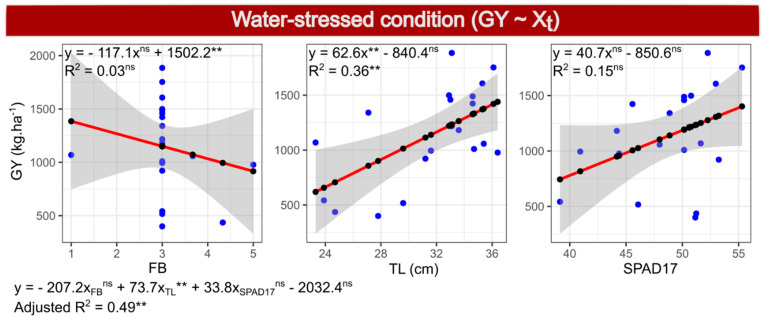
Simple linear regressions (within boxes) and multiple regressions (below the boxes) between the dependent variable grain yield (GY) and independent variables leaf rolling (FB), tassel length (TL) and SPAD index, measured 17 days after anthesis (SPAD17), evaluated in 20 popcorn inbred lines under water stress.

In the simple regressions for each predictor variable, only FB had a negative coefficient but the highest value (Figure 2 and Figure 6). Although the coefficient of FB was the highest, the R^2^ value of FB in simple linear regression was far lower (0.03) than that for TL (0.36), and the *p*-value was 0.40, whereas TL had *p* = 0.005. Variable SPAD17 also had a low R^2^ value, with an estimate of 0.15 and *p* = 0.08.

### 2.8. Selection of Model 2—GY under WW Conditions

In Model 2 for GY, under WW conditions, the predictor variables SPAD17 and TL were selected, with reduction of the Akaike value from 251 in the initial to 250 in the final model, with an adjusted R^2^ of 0.62 and *p* = 0.001 (Final Model 2: GY~TL + SPAD17) and significance of both variables.

**Figure 3:**
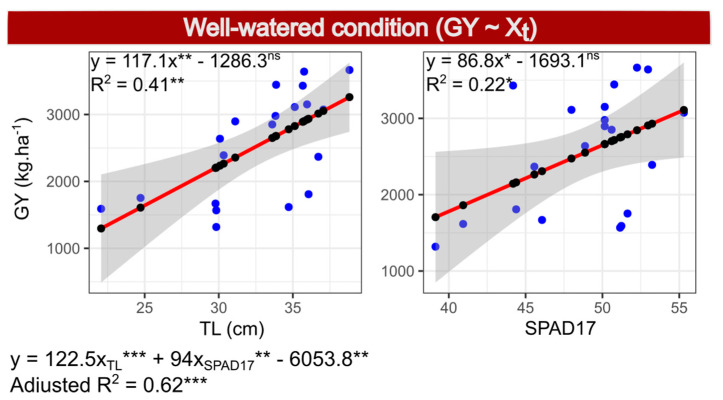
Simple linear regressions (within boxes) and multiple regressions (below the boxes) between the dependent variable grain yield (GY) and independent variables tassel length (TL) and SPAD index, measured 17 days after anthesis (SPAD17) and evaluated in 20 popcorn inbred lines under well-watered conditions.

In the simple linear regression, the R^2^ of SPAD17 was 0.22 and the *p*-value 0.037, while TL had a R^2^ of 0.41 and *p* = 0.002. Both variables had positive coefficients, with no significant differences between each other (Figure 3 and Figure 6).

### 2.9. Selection of Model 3—PE under WS

**Figure 4:**
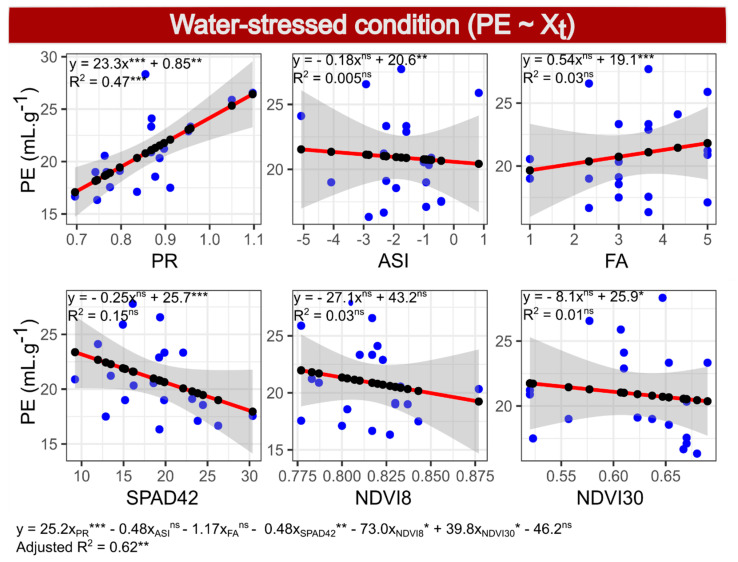
Simple linear regressions (within boxes) and multiple regressions (below three boxes) between the dependent variable popping expansion (PE) and independent variables: prolificacy (PR), anthesis-silking interval (ASI), leaf angle (FA), SPAD index measurement 42 days after anthesis (SPAD42) and normalized difference vegetation indexes evaluated 8 (NDVI8) and 30 (NDVI30) days after anthesis, evaluated in 20 popcorn inbred lines under water stress.

In Model 3, considering the dependent variable PE under WS, the variables selected by the Akaike criterion were PR, ASI, FA, SPAD42, NDVI8 and NDVI30, with a reduction in the Akaike value from 39.26 to 35.70 in the final model, with an adjusted R^2^ of 0.62 and *p*-value of 0.003 (Final Model 3: PE~PR + ASI + FA + SPAD42 + NDVI8 + NDVI30). In the selected model, the traits PR, SPAD42, NDVI8 and NDVI30 were significant by the *t*-test, with a positive sign of the coefficients of PR and NDVI30, while the others had a negative sign. However, in a simple regression between NDVI30 and PE, no significance for the model or the predictor was found, and the slope was negative (Figure 4 and Figure 6). In addition, only the PR showed model significance by a simple regression (*p* < 0.001), with an estimated R^2^ of 0.47. Variable SPAD42 had *p* = 0.09 and R^2^ of 0.15, and the other variables *p* > 0.4 and R^2^ estimates were close to zero.

### 2.10. Selection of Model 4—PE under WW Conditions

**Figure 5:**
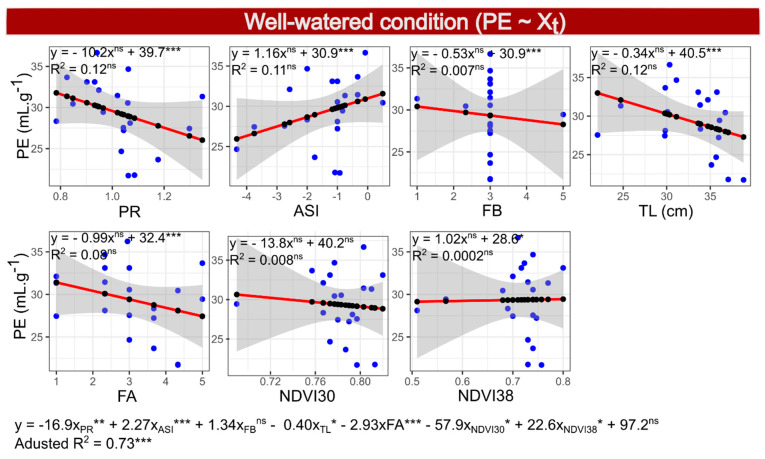
Simple linear regressions (within boxes) and multiple regressions (below the boxes) between the dependent variable popping expansion (PE) and independent variables prolificacy (PR), anthesis-silking interval (ASI), leaf rolling (FB), tassel length (TL), leaf angle (FA) and normalized difference vegetation index evaluated at 30 (NDVI30) and 38 (NDVI38) days after anthesis, evaluated in 20 popcorn inbred lines under well-watered conditions.

**Figure 6:**
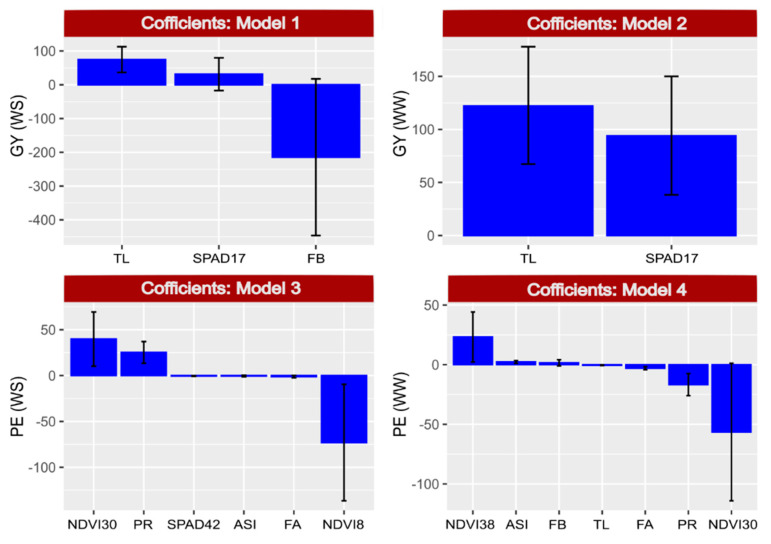
Coefficient related to the effects of previously selected predictors on the dependent variables. Model 1: grain yield (GY) under water stress (WS) = TL (tassel length) + SPAD17 (SPAD index evaluated 17 days after anthesis) + FB (leaf rolling). Model 2: GY under well-watered conditions (WW) = TL + SPAD17. Model 3: popping expansion (PE) under WS = NDVI30 (normalized difference vegetation index evaluated 30 days after anthesis) + PR (prolificacy) + SPAD42 (evaluated 42 days after anthesis) + ASI (anthesis-silking interval) + FA (leaf angle) + NDVI8 (evaluated 8 days after anthesis). Model 4: PE under WW = NDVI38 (evaluated 38 days after anthesis) + ASI + FB + TL + FA + PR + NDVI30.

In Model 4, for the dependent variable PE under WW, the traits PR, ASI, FB, TL, FA, NDVI30 and NDVI38 were selected by the Akaike criterion, with a reduction from 38.1 to 36.2, from the initial to the final model (final model 4: PE~PR + ASI + FB + TL + FA + NDVI30 + NDVI38). The adjusted R^2^ of the final model 4 was 0.73, with a *p*-value of 0.0007, indicating a good chance of predicting the PE. Considering the variables in the multiple regressions, only FB was not significant, and FB, ASI and NDVI38 had positive, and the others negative, coefficients. On the other hand, considering the isolated effect of the predictors in simple regressions, only ASI and NDVI38 were positively related with PE (Figure 5 and Figure 6). However, the model was not significant for any of the predictor variables, and R^2^ was lower than 0.21 in simple regressions, requiring the simultaneous use of all selected variables to ensure an efficient selection, increasing the complexity of the selection based on many predictors.

### 2.11. Predictive Effect on Dependent Variables (GY and PE) and Relative Importance of Independent Variables

**Figure 7:**
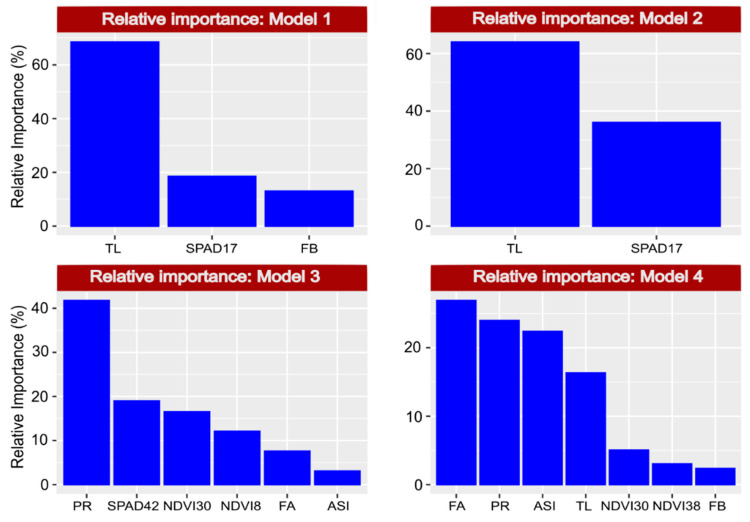
Relative importance of previously selected predictors on the basis of the dependent variable. Model 1: grain yield (GY) under water stress (WS) = TL (tassel length) + SPAD17 (SPAD index evaluated 17 days after anthesis) + FB (leaf rolling). Model 2: GY under well-watered conditions (WW) = TL + SPAD17. Model 3: popping expansion (PE) under WS = NDVI30 (normalized difference vegetation index evaluated 30 days after anthesis) + PR (prolificacy) + SPAD42 (evaluated 42 days after anthesis) + ASI (anthesis-silking interval) + FA (leaf angle) + NDVI8 (evaluated 8 days after anthesis). Model 4: PE under WW = NDVI38 (evaluated 38 days after anthesis) + ASI + FB + TL + FA + PR + NDVI30.

The coefficients in Figure 6 are related to the effect of the predictor on the dependent variable, while the relative importance (Figure 7) indicates the mean level of importance of each variable in the adjustment to the model due to its association with the dependent variable.

Variable TL, present in Model 1 (GY under WS), Model 2 (GY under WW) and Model 3 (PE under WS), had a high relative importance in all three models, with a coefficient of 74.5 and 122 in Models 1 and 2, respectively, and a coefficient of −0.4 in Model 4 (PE under WW conditions) (Figure 6). In the models of the dependent variable PE (3 and 4), the evaluations of the NDVI detected the highest coefficients with low relative importance. On the other hand, PR had a high relative importance but high coefficients in both models (Figure 7). The variables FA and ASI also had high relative importance, whereas the coefficients were low (Figure 6).

**Figure 8:**
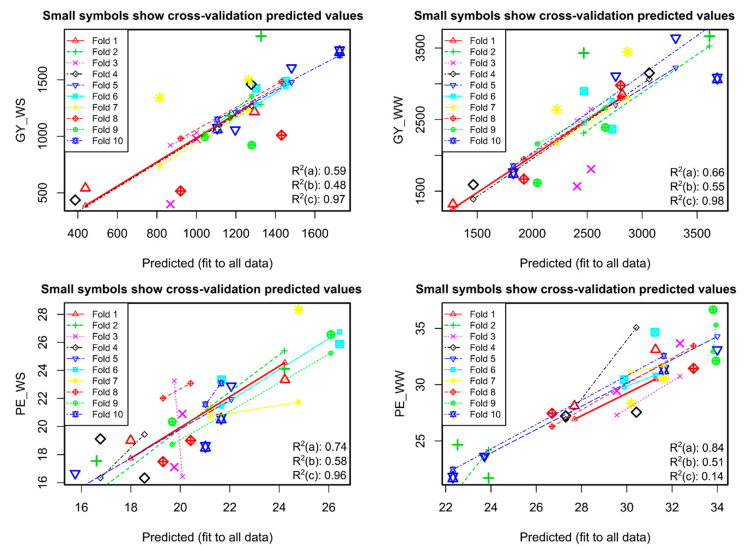
Regressions based on the predicted values of 10-fold cross-validation in each of the four models selected for the response variables: GY_WS (grain yield under water stress), GY_WW (grain yield under well-watered conditions), PE_WS (popping expansion under water stress) and PE_WW (popping expansion under well-watered conditions). The R^2^ values corresponded to the coefficients of determination between: (a) observed versus adjusted values, (b) observed values versus values adjusted by the folds and (c) adjusted values versus values adjusted by the folds.

In the cross-validation of models using 10 folds (k = 10), the reliability of the models with GY as the response variable was greater than for the models with PE. In Model 1, the R^2^ values were (i) 0.59 for values observed with the adjusted, (ii) 0.48 between the observed values and adjusted values from the folds and (iii) 0.97 between the adjusted values and those adjusted to the samples from the folds. In Model 2, the R^2^ values were 0.66, 0.55 and 0.98 following the same order. In Model 3, they were 0.74, 0.58 and 0.96 and 0.84, 0.51 and 0.14, respectively, in Model 4 (Figure 8).

## 3. Discussion

### 3.1. Effect of Water Restriction on GY, PE, Morpho-Agronomic Traits and on the Greenness Index

Grain yield and popping expansion were the traits most affected by water restriction in the soil, with reductions of 55% and 28%, respectively. According to Kamphorst et al. (2020) [20], in response to WS applied during the pre-flowering and grain filling of popcorn, the effect of reduction on yield covariates was weaker, e.g., on number of grains per row, grain rows per ear and ear diameters and lengths. However, the sum of these effects drastically reduced the main variables, GY and PE. For field corn under water stress, a reduction in the number of grains produced per area is associated with a lower GY [17]. For popcorn, the reduction is due to a lower number of grains produced per ear, as well as a lower 100-grain weight [21]. Certainly, the pollen viability and zygote formation are sensitive to water deficit [38,39], reducing the number of grains produced, as confirmed in the cited cases.

Marked reductions of more than 10% were observed for the traits 100-grain weight and prolificacy. When water restriction occurred during grain filling, a reduction in grain weight was observed, and WS before anthesis decreased the number of grains [20] due to the effect of water restriction on pollen viability and zygote formation [38,39]. During maize development, the number of ears per plant (prolificacy) was determined in stages V6–V8 [27,40]. Thus, for this trait, the weakest effect of the water deficit was expected when the stress occurred during grain filling. In this study, the ear was already formed when water restriction was applied, and some plants produced no grain due to the water deficit in the plant, which is why the reduction in the mean PR was not assessed.

A tendency to extend the anthesis-silking interval (ASI) in response to water stress was also observed in this study. This observation is frequently reported for maize plants [26,41] and relevant for genetic dissection, given the strong correlation between ASI and maize grain yield under dry conditions [1]. It is well-known that drought-induced ASI hinders successful pollination, negatively impacting grain production [1,38]. In field corn and popcorn plants, the asynchrony of male and female flowering characterizes a mechanism of “drought escape”, by which the cycle is adjusted to return to adequate conditions [1,42].

Under dry conditions, plants tend to have a greater leaf angle in relation to the stalk (smaller angle in relation to the soil surface), as well as intensified leaf rolling. Under dry soil conditions, Baret et al. (2018) [43] observed that leaf rolling makes the leaves stiffer and more erect in relation to the stalk. This phenomenon (angular change) occurs to reduce the surface area exposed to the sun. In this study, leaf rolling was observed in plants under WS but was not measured. Since FA and FB were evaluated in the period of physiological maturity, near the harvest, the advanced plant senescence impeded the observation of the phenomenon described by Baret et al. (2018) [43]. In fact, in this study, the plants under WS had a wider angle (FA) and stronger leaf rolling (FB) due to exposure to greater solar radiation and less turgor due to stomatal closure under the higher incidence of solar radiation [1,27]. Clearly, leaf senescence was more marked under WS (Table 1).

Leaf senescence is governed by the different phenological stages of plants but is also strongly influenced by the environment [44]—in particular, by water restriction. Both at the leaf level—measured by the SPAD index—and the canopy level—measured by the NDVI—leaf senescence occurred earlier in the genotypes evaluated under WS. In this study, where irrigation was suspended before male flowering (15 days before flowering) and based on the SPAD index, it was observed that water-stressed plants did not reach the maximum greenness index; in other words, plants under WW conditions had a mean SPAD index of 49.08, while, for plants under WS, the mean was 46.23. These values were assessed seven days after male flowering. On the other hand, in an evaluation of the canopy, eight days after male flowering, these differences were undetectable in a comparison of the estimates of 0.81 and 0.82, respectively, observed under WW and WS.

The drought escape mechanism, i.e., the crop completes the cycle before it is harmed by drought effects, was also noted in an accelerated senescence of the popcorn plants at the leaf and canopy levels. The greenness index, measured by the SPAD index and NDVI, allowed inferences about the photochemical machinery, since these values were strongly and positively correlated with the leaf chlorophyll concentration [3,45,46]. Thus, due to the marked reduction in the values of the SPAD index and NDVI under WS, the photosynthetic carbon assimilation may have been reduced, which may have decreased the dependent variables GY and PE.

The variables of economic interest, GY and PE, were significantly affected by the G*WC interaction, indicating differentiated responses of the evaluated genotypes to the different WCs. Interactions of this nature jeopardize gains by selection and the recommendation of cultivars for specific environments [47]. Based on this observation, multiple regression analyses were performed for each WC-dependent variable.

### 3.2. Multiple Regressions

Multiple linear regression is a special case of linear regression by which the relationship between a response variable and two or more independent variables and their interactions are analyzed [48]. In the first part of this study, the evaluation of multiple predictors (19 variables) served to exclude those whose removal caused no significant loss of information (so-called extraneous terms). In all scenarios of this study, the traits GW, TB, SPAD7, SPAD12, SPAD22, SPAD28, SPAD35, NDVI19 and NDVI24 were considered unnecessary for the prediction of GY or PE under WS or WW conditions. Furthermore, the subdivision of variables increased the observation: the variable ratio. At this moment, important variables frequently used as selective for higher GY in maize were excluded, e.g., TB [13,27] and those related to stay-green [13,24,25].

The significance of FB for the prediction of GY and PE under WS (Table 1 and Figure 1) suggests the influence of this characteristic under WS; however, with no clear effect under the WW conditions. The association between the variables had a negative sign. This indicated that the plants with more erect leaf rolling (score 1) were those with higher GY and PE. Plants with slight or strong FB (scores 3 or 5) have a larger area of direct sunlight incidence on the leaves [27] and consequently, under water restriction, the leaf temperature rises, leading to stomatal closure, intensifying the production of reactive oxygen species (ROS) and, finally, accelerating the degradation of chlorophyll molecules [1,43]. Despite these observations and the biological relevance, in the model selection using the Akaike criterion, FB was disregarded for the prediction of PE under WS, where this trait played an important role in the prediction of GY under drought. On the other hand, considering another set of variables, FB was selected in Model 4 (PE under WW); however, with a low coefficient, low relative importance and low R^2^ in simple regression, indicating an ineffectiveness in indirect selection, FB is therefore recommended to be used separately.

Models 1 and 2 of the GY prediction under WS and WW, respectively, were more successful than Models 2 and 3 due to the higher R^2^, greater significance and for being more parsimonious (lower number of elements). The coefficients of the two predictors in common to both models (TL and SPAD17) were positive in multiple and simple regressions. Of the two, TL had a greater relative importance under both WCs (Figure 7), and the R^2^ in the simple regression was significant. With a view toward WS tolerance, a shorter tassel with a lower number of branches is desirable [13]. Some studies have suggested that a larger tassel size and higher number of tassel branches tend to reduce the grain yield due to the decrease in sunlight interception by the flag leaves (self-shading) and the competition for photoassimilates for different plant structures [49]. Despite the above, for the evaluated popcorn inbred lines, the trait proved relevant for yields under WS and WW conditions and was positively correlated with GY.

For the assessments of SPAD and NDVI, it is worth mentioning that the efficiency of the measurement methodologies of the greenness index partly resides in an application at the stages critical for grain production [15,17]. In this study, measuring the greenness index based on SPAD 17 days after male flowering proved to be appropriate to predict the GY values.

On the other hand, trait selection based on simple linear models for PE prediction under the WS and WW conditions proved ineffective; only PR was significant and had an acceptable R^2^ value (0.44) under WS (Model 3). In turn, considering the multiple models for PE, under both WCs, the significances and adjusted determination coefficients of the models were high, albeit very complex, due to the excess of explanatory variables, making their use unfeasible. Conversely, indirect selection from the variables selected for GY prediction, such as TL and SPAD17, may possibly be used with no major negative influence on PE. Commonly, the genetic correlation of PR with GY is high, both under normal [50] as well as stress conditions [26]. Some studies have shown that, under water restrictions, the heritability for PR remained constant or even increased, opposite to the observations for grain yield [49], which may favor genetic gains by indirect selection for this trait.

The use of multiple traits instead of a single characteristic in indirect selection improved the adjustment to the regression in this study, which could increase the gains for the dependent variables. Among the different frequently used traits for the selection of more water stress-tolerant maize plants, those described and evaluated in this research indicated that a larger tassel size was important for GY predictions. In popcorn, plants with higher yields had a larger tassel size, whereas the opposite was observed for field corn plants, for which smaller tassels are recommended for an efficient and successful selection. With regard to senescence, however, similar to the field corn, the evaluation of individual leaves around 15–20 days after male flowering seem to be important to predict higher popcorn GY.

## 4. Materials and Methods

Twenty S_7_ popcorn inbred lines from germplasms derived from countries in tropical (L61, L63, L65, L69, L70 and L71—from the “BRS-Angela” population) and temperate (P1, P5 and P7—from the hybrid “Zélia”; P2 and P3—from the compound “CMS-42”; P4—from South American breeds; P6, P8 and P9—from the hybrid “IAC-112”; L54, L55 and L59—from the population “Beija Flor” and L75 and L76—from the population “Barão de Viçosa”) regions of South America [51] were experimentally evaluated in the northern region of Rio de Janeiro, Brazil (21°42′48″ S, 41°20′38″ W; 14 m asl). The soil of the experimental station, with high clay and silt contents, was classified as Argissolo Amarelo Distrófico Fragipânico Latossólico. The trials were carried out in the dry period of the growing season of 2016 from April to August, with popcorn sowing on 10 April and harvest on 15 August.

Fertilization was applied as recommended for the crop [52] based on a soil report. In summary, 30-kg N ha^−1^, 60-kg P_2_O_5_ ha^−1^ and 60-kg K_2_O ha^−1^ were applied at planting and 100-kg N ha^−1^ as top dressing 30 days after sowing (DAS). The experimental plots consisted of four 4.40-m rows, spaced 0.20 m between plants and 0.80 m between rows (62.500 plants ha^−1^).

The experiment was arranged in a randomized complete block design with three replications under two contrasting water conditions (WC): well-watered (WW) and water-stressed (WS) conditions. Under WW conditions, the field capacity (−10 kPa) was maintained by irrigation and monitored with tensiometers (MPS-6, Decagon Devices, Pullman, WA, USA) while, under WS, irrigation was stopped at the phenological stage before male flowering, 49 days after sowing and 15 days before male flowering, and the permanent wilting point (−1500 kPa) was reached 12 days after male flowering (data not shown). The crop was irrigated by one Katif-type dripper per plant at a flow rate of approximately 2.3 mm h^−1^.

Precipitation, temperature, air humidity and solar radiation (Figure S1A,B) were measured at a meteorological station of the National Institute of Meteorology (INMET), installed at the experimental station. In summary, during the experimental period, the total rainfall was 133 mm, the temperature and relative humidity varied between 12 and 37 °C and 23% and 97%, respectively, with a mean solar radiation of 20.35 MJ m^−2^ day^−1^ (photosynthetically active radiation (PAR) ≅ 1300 µmol m^−2^ s^−1^). Under WS, the plants were irrigated with 60 mm, while, for full saturation, an additional amount of water was provided, resulting in a total of 138 mm (Figure S1A).

### 4.1. Traits Evaluated

The evaluated traits were grouped as related to: (i) response or dependent variables (y)—namely, grain yield (GY) and popping expansion (PE); (ii) morpho-agronomic traits—namely, 100-grain weight (GW), prolificacy (PR), tassel length (TL), number of tassel branches (TB), anthesis-silking interval (ASI), leaf angle (FA) and leaf rolling (FB); (iii) the greenness index measured by a portable chlorophyll meter SPAD and (iv) functional/structural properties of the canopy, based on the normalized difference vegetation index (NDVI). Groups iii and iv were measured on different dates during the grain-filling period.

The dependent variable GY was determined by weighing the grains of each plot, corrected to a moisture content of 13% and extrapolated to kg ha^−1^. The variable popping expansion (PE) was measured by quantifying the popcorn volume of 30 g of grains microwaved (180 °C) in a kraft paper bag for 2 min. The popcorn volume, quantified in a 2000-mL beaker, was divided by the popcorn kernel weight (30 g) and expressed in mL g^−1^.

The following morpho-agronomic traits were evaluated: GW, determined by the mean weight (g) of two samples of 100 grains per plot; PR, calculated as the quotient of the number of ears harvested by the total number of plants per plot; TL, measured with a ruler (cm), from the main branch to the tip of the tassel; TB, determined by counting the tassel branches; ASI, calculated as the difference between the mean values on the dates (days) of male and female flowering, and FA and FB, which were evaluated using illustrative figures from the International Plant Genetic Resources Institute [53], in which leaf angles were scored (1, 3 and 5 for small, medium and large, respectively) and leaf rolling (1, 3 and 5 for straight, curved and strongly curved, respectively. The traits GW, PR and ASI were measured based on all plants of a plot and TL, TB, FA and FB on a random sample of six plants per plot.

At the leaf level (SPAD index), the “greenness” index was estimated based on three readings on each evaluation date, in a random sample of six plants per plot, by assessing the middle-third of the third leaf counted from the tip and below the flag leaf, with a portable chlorophyll meter SPAD 502 (Minolta, Tokyo, Japan). The SPAD index of a random sample of six plants per plot was measured on seven different dates (days after anthesis; DAA) in relation to the reference event male flowering, namely: in seven DAA (SPAD7), 12 DAA (SPAD12), 17 DAA (SPAD17), 22 DAA (SPAD22), 28 DAA (SPAD28), 35 DAA (SPAD35) and 42 DAA (SPAD42).

At the canopy level, the normalized difference vegetation index (NDVI) was measured with an optical sensor, FieldScout CM 1000 NDVI (Spectrum Technologies, Aurora, IL, USA). The equipment has two sensors, one in the visible (660 nm) and the other in the infrared region (840 nm). The software calculates the NDVI according to the equation: NDVI = [(% Near Infrared − % Red)/(% Near Infrared + % Red)], where % Near Infrared is the % of infrared reflectance (840 nm) of the leaves, and % Red is the leaf reflectance at a wavelength of 660 nm. The NDVI values range from −1 to 1. At values close to 1, the metabolic and physiological functions of the leaves are in full use, while values close to 0 indicate impaired or suspended metabolic and physiological functions of the vegetation (Adebayo et al., 2014). Once set in motion by hand, the optical sensor CM 1000 was run along the two central rows of each plot, from one end to the other, at a height of about 60 cm above the plant canopy. The NDVI was measured on five different dates, which were based on male flowering, namely: 8 (NDVI8), 19 (NDVI19), 24 (NDVI24), 30 (NDVI30) and 38 (NDVI38) days after anthesis.

### 4.2. Analysis of Variance for Each Water Condition (WC) and Combined Analysis

The proportional reduction (%) of each variable, given the comparison between the WC, was calculated as: $100-\left[\left(Y_{WS}/Y_{WW}\right)*100\right]$, where Y is the overall mean of the variable WS and WW conditions.

### 4.3. Multiple Linear Regressions (MLR)

The first part of the study refers to a selection of variables analyzed in three groups of characteristics, namely: morpho-agronomic (MORPH) and leaf greenness indices, estimated based on SPAD and the NDVI, at different dates after male flowering. Each set of characteristics was included in the model as a parametric predictor of the response variables (GY and PE) under two water conditions (WW and WS). On this basis, 12 multiple linear regressions were performed (3 trait groups × 2 water conditions × 2 response variables). The model: yi = α + x_i,1_β_1_ + x_i,2_β_2_ +... + x_i,p_β_p_ + ε_i_ was used for the sets of variables measured under both WCs for both GY and PE, where α is the unknown model constant (intercept), xi the p × 1 vector of predictor variables of each trait group, β the p × 1 vector of the coefficients and ε the random error.

With the 12 regressions, the characteristics that were significant under at least one of the water conditions (WC) were selected and then grouped as predictors of the dependent variables (GY and PE). A PCA-biplot was constructed with these predictor and dependent variables to facilitate the understanding of the behavior of the variables under each WC. To avoid multicollinearity problems between the predictor candidate variables, the inflation of the regression coefficients due to multicollinearity was checked with the variance inflation factor, eliminating predictors with values close to or greater than 10 [37]. Variables without multicollinearity problems were reanalyzed in the multiple linear regression models, grouping the morpho-agronomic, SPAD and NDVI variables separately for each WC, and, finally, the best models were selected by the Akaike criterion using the stepwise selection method, which consists of adding and removing predictors.

The relative importance of the predictors of each model was estimated by the method lmg (acronym for Lindeman, Merenda and Gold) [54] based on the mean of p! orders of predictor inclusion and increment of R^2^, where *p* is the number of predictors in the model.

The resulting models were subjected to a k-fold cross-validation, in which datasets were randomly assigned by removing one fold per round, at *k* = 10, and the remaining data were used to adjust a new regression model that served to predict the excluded observations. For the k-fold cross-validation, we used the estimator $CV_{\left(K\right)}=\overset{K}{\underset{k=1}{\sum }}\frac{n_{k}}{n}\;EQM_{k}$, where *K* represents the number of folds, *n_k_* the number of observations of the *k*th partition and EQMk=∑i∈Ck(yi−yi^)²nk , where $\hat{y_{i}}$ is the adjusted value of observation *i*, derived from the data without the *k*th part, and Ck represents the index of the *k*th part [55].

The analyses were performed with R software [56].

## 5. Conclusions

The grain yield proved to be a more appropriate characteristic for indirect selection based on multiple predictors. For the indirect selection based on a single predictor, tassel lengths and the SPAD index measured 17 days after male flowering were efficient for grain yield selection under WS, while the variable prolificacy was efficient for indirect selection for popping an expansion under drought conditions. The exploitation of these traits by indirect selection is expected to induce increments in grain yield and popping expansion.

## Figures and Tables

**Table 1:** Summary of individual and combined analyses of variance and mean estimates, standard deviations and proportional reductions under two water conditions (WS and WW conditions) of the dependent variables GY and PE and of the morpho-agronomic and physiological traits (SPAD and NDVI) evaluated on different measurement dates in popcorn inbred lines.

Traits	WC	Mean ± Standard Deviations			Proportional Reductions (%)	Interaction G*WC
Dependent variables						
GY	WS	1139.11	±	477.24	55.30	*
	WW	2548.07	±	910.89		
PE	WS	20.91	±	3.74	28.75	**
	WW	29.35	±	5.24		
Morphological traits						
GW	WS	9.70	±	1.47	23.49	ns
	WW	12.67	±	1.71		
PR	WS	0.86	±	0.14	15.84	ns
	WW	1.02	±	0.18		
TL	WS	31.60	±	4.56	3.50	ns
	WW	32.75	±	4.62		
TB	WS	14.18	±	3.48	7.01	ns
	WW	15.25	±	4.61		
ASI	WS	−1.77	±	1.41	−25.44	ns
	WW	−1.41	±	1.29		
FA	WS	3.33	±	1.34	−8.70	ns
	WW	3.07	±	1.38		
FB	WS	3.10	±	0.77	−4.49	ns
	WW	2.97	±	0.69		
SPAD index						
SPAD7	WS	46.23	±	4.03	5.82	ns
	WW	49.08	±	3.47		
SPAD12	WS	45.47	±	4.47	4.48	ns
	WW	47.61	±	4.12		
SPAD17	WS	43.55	±	4.17	10.82	ns
	WW	48.84	±	4.55		
SPAD22	WS	40.33	±	5.15	17.64	ns
	WW	48.97	±	4.15		
SPAD28	WS	39.70	±	5.15	17.56	ns
	WW	48.16	±	3.97		
SPAD35	WS	32.51	±	6.91	29.32	ns
	WW	45.99	±	5.17		
SPAD42	WS	18.81	±	6.60	52.20	ns
	WW	39.36	±	6.73		
Normalized Difference Vegetation Index (NVDI)						
NDVI8	WS	0.82	±	0.03	−0.20	ns
	WW	0.81	±	0.04		
NDVI19	WS	0.73	±	0.04	9.51	ns
	WW	0.81	±	0.04		
NDVI24	WS	0.67	±	0.05	15.28	ns
	WW	0.79	±	0.04		
NDVI30	WS	0.62	±	0.07	20.41	ns
	WW	0.78	±	0.04		
NDVI38	WS	0.47	±	0.10	33.26	ns
	WW	0.71	±	0.10		

Morphological traits: GW = 100-grain weight (g), PR = prolificacy (unit), TL = tassel length (cm), TB = number of tassel branches (unit), ASI = anthesis-silking interval (days), FA = leaf angle (score) and FB = leaf rolling (score). Physiological traits: SPAD index (SPAD 7–42) = SPAD index at 7,12, 17, 22, 28, 35 and 42 days after male flowering, respectively, and the Normalized Difference Vegetation Index (NDVI 8–38) = Normalized Difference Vegetation Index at 8, 19, 24, 30 and 38 days after male flowering, respectively. G*WC: interaction genotype * water condition. **, * and ns: significant at 1% and 5% probability and nonsignificant, respectively, by the F test.

**Table 2:** Estimated beta values using multiple regression based on seven morphological (MORPH) traits (predictors) versus grain yield (GY) and popping expansion (PE) (dependent variables) of 20 popcorn inbred lines evaluated under two water conditions.

Variables	GY~MORPH		PE~MORPH	
	WS	WW	WS	WW
Intercept	−2105.89	−3035.85	−3.32	66.02 ***
GW	100.82	215.02	0.06	−0.03
PR	604.33	−1143.68	33.17 **	−17.33 *
TL	68.61 *	135.02 *	−0.32 *	−0.37
TR	23.99	−2.505	0.38	−0.029
ASI	25.93	−98.29	−0.42	1.81 *
FA	95.55	−1.66	0.51	−2.55 **
FB	−333.45 *	−164.81	−0.79 *	1.55
Adjusted R^2^	0.52 *	0.31 ^ns^	0.39 ^ns^	0.57 **

WS = water stress, WW = well-watered, GW = 100-grain weight, PR = prolificacy, TL = tassel length, TB = number of tassel branches, ASI = anthesis-silking interval, FA = leaf angle and FB = leaf rolling. *, **, *** and ns indicate significance at 5%, 1% and 0.1% probability and no significance, respectively, by the *t*-test for the variables and the F test for the models.

**Table 3:** Estimated beta values using multiple regression based on seven predictors of the SPAD index (measured 7, 12, 17, 22, 28, 35 and 42 days after male flowering) versus the dependent variables grain yield (GY) and popping expansion (PE) evaluated in 20 popcorn inbred lines under two water conditions (WCs).

Variables	GY~SPAD Index		PE~SPAD Index	
	WS	WW	WS	WW
Intecept	778.45	6334.46	43.52626 **	15.88745
SPAD7	27.12	−190.49	−0.56	0.2992
SPAD12	−45.5	−119.97	−0.26	0.118
SPAD17	−37.47	280.58 **	0.42	−0.59819
SPAD22	81.88	37.61	−0.03	−0.37379
SPAD28	−57.05	23.85	−0.13	0.65234
SPAD35	78.86	−216.18	0.49	0.18314
SPAD42	−42.14	114.95	−0.61582 *	0.02108
Adjusted R^2^	0.2257 ^ns^	0.425 *	0.1937 ^ns^	−0.4308 ^ns^

WS = water-stressed, WW = well-watered, SPAD index at 7–42 = SPAD index at 7, 12, 17, 22, 28, 35 and 42 days after male anthesis, respectively. *, ** and ns correspond to the significance at the levels of 5% and 1% of probability and no significance in the *t*-test for the variables and the F test for the models.

**Table 4:** Estimated beta values using multiple regression based on five Normalized Difference Vegetation Index (NDVI) measurements at 8, 19, 24, 30 and 38 days after male flowering (predictors) versus the grain yield (GY) and popping expansion (PE) (dependent variables) evaluated in 20 popcorn inbred lines under two water conditions (WCs).

Variable	GY~NDVI		PE~NDVI	
	WS	WW	WS	WW
Intecept	3667	−4247	66.72 *	−13.99
NDVI8	−6149	1950	−18.86	100.31 *
NDVI19	8744	−8872	3.32	116.93
NDVI24	−7108	15358	−84.78	−26.73
NDVI30	1965	−4620	64.31 *	−154.04 *
NDVI38	−894	5550	−34.50 *	11.75
Adjusted R^2^	0.233 ^ns^	0.076 ^ns^	0.268 ^ns^	0.268 ^ns^

WS = water-stressed, WW = well-watered, NDVI at 8–38 = Normalized Difference Vegetation Index at 8, 19, 24, 30 and 38 days after male anthesis, respectively. * and ns corresponds to the significance at the levels of 5% of probability and no significance by the *t*-test for the variables and the F test for the models.

## Supplementary Materials

The following are available online at https://www.mdpi.com/article/10.3390/plants10081510/s1: Figure S1: (A) Rainfall during a trial with 20 popcorn lines under well-watered and water-stressed conditions. (B) Air humidity, radiation and temperature values, measured under water-stressed and well-watered conditions. Figure S2: Distribution error distribution of the four selected models for the four response variables: Model 1: GY~WS~FIT~Sel (grain yield under water stress), Model 2: GY~WW~FIT~Sel (grain yield under well-watered conditions), Model 3: PE~WS~FIT~Sel (popping expansion under water stress) and Model 4: PE~WW~FIT~Sel (popping expansion under well-watered conditions).
