# Supplementary material for: Supporting Physiological Trait for Indirect Selection for Grain Yield in Drought-Stressed Popcorn
Source: Plants (Basel). 2021 Jul 23;10(8):1510. doi: 10.3390/plants10081510 (PMC8399448; doi:10.3390/plants10081510)
Supplement: Supplementary file 1 [file plants-10-01510-s001.zip › plants-1305665-supplementary.pdf]

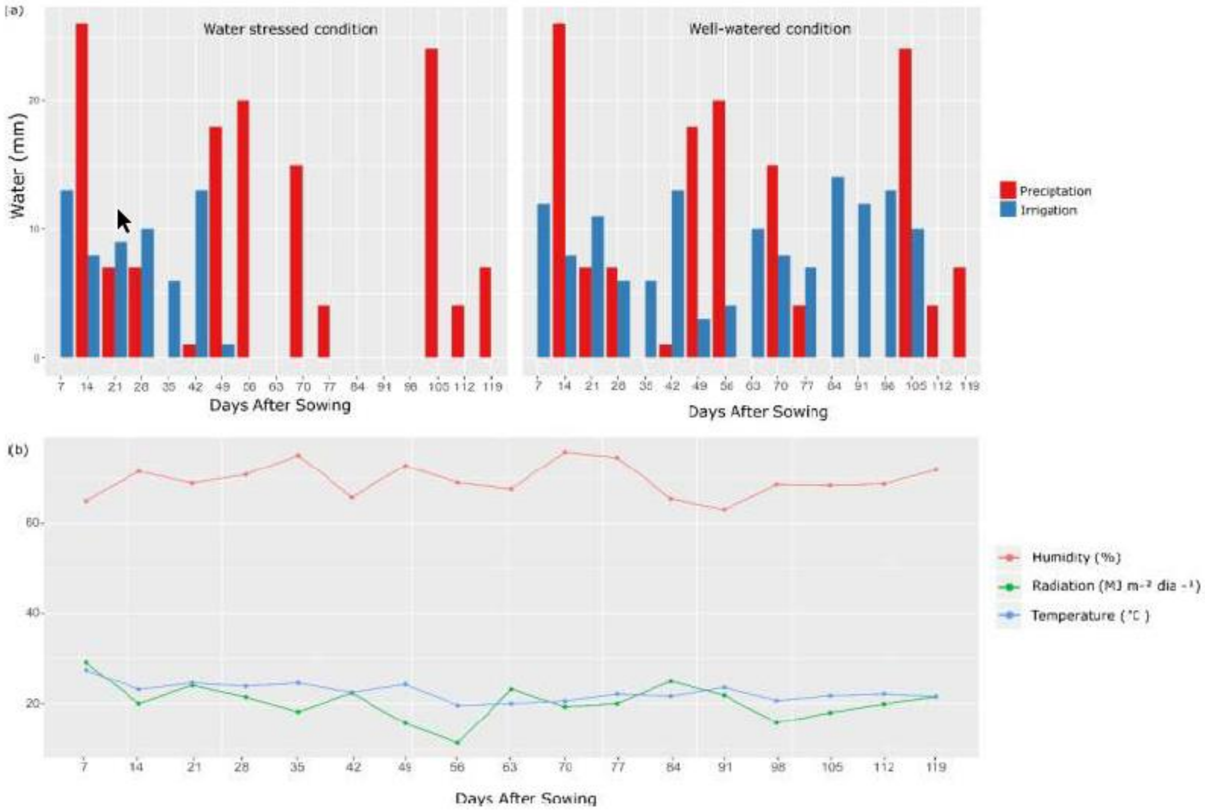

Figure S1. (a) Rainfall during a trial with 20 popcorn lines under well-watered and water-stressed conditions. (b) Air humidity, radiation and temperature values, measured under water-stressed and well-watered conditions.

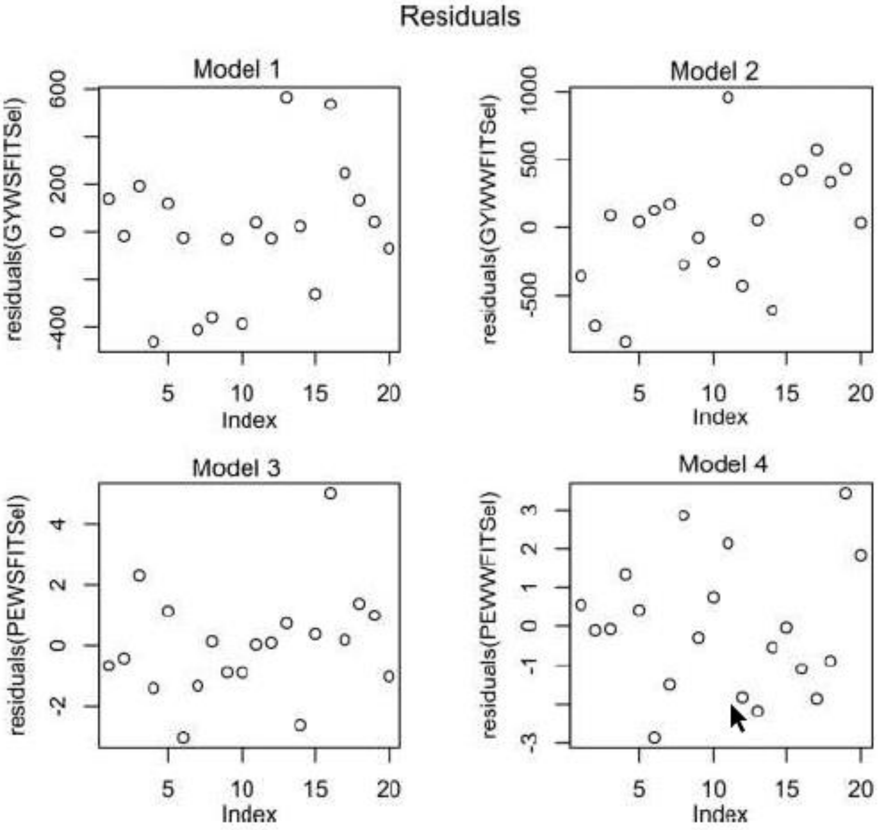

Figure S2. Distribution error distribution of the four selected models for the four response variables: Model 1: GY~WS~FIT~Sel (grain yield under water stress), Model 2: GY~WW~FIT~Sel (grain yield under well-watered conditions), Model 3: PE~WS~FIT~Sel (popping expansion under water stress) and Model 4: PE~WW~FIT~Sel (popping expansion under well-watered conditions).
